# 3-(2,4-Dichloro­phen­yl)-5-phenyl-1,2,4-oxadiazole

**DOI:** 10.1107/S160053681000927X

**Published:** 2010-03-17

**Authors:** Hoong-Kun Fun, Mohd Mustaqim Rosli, Sankappa Rai, Arun M. Isloor, Prakash Shetty

**Affiliations:** a X-ray Crystallography Unit, School of Physics, Universiti Sains Malaysia, 11800 USM, Penang, Malaysia; b Syngene International Ltd, Biocon Park, Plot Nos. 2 & 3, Bommasandra 4th Phase, Jigani Link Rd, Bangalore 560 100, India; c Department of Chemistry, Organic Chemistry Division, National Institute of Technology-Karnataka, Surathkal, Mangalore 575 025, India; d Department of Printing, Manipal Institute of Technology, Manipal 576 104, India

## Abstract

In the title compound, C_14_H_8_Cl_2_N_2_O, the dihedral angles between the plane of the oxadiazole ring and those of the benzene rings are 2.3 (1) and 9.5 (1)°. In the crystal, mol­ecules are linked into chains along the *c* axis by C—H⋯Cl inter­actions. These chains are stacked along the *a* axis.

## Related literature

For the biological properties of heterocyclic compounds including oxadiazo­les, see: Andersen *et al.* (1994 ▶); Showell *et al.* (1991 ▶); Watjen *et al.* (1989 ▶); Swain *et al.* (1991 ▶); Clitherow *et al.* (1996 ▶). For their pharmacological and medicinal activity, see: Isloor *et al.* (2010 ▶); Chandrakantha *et al.* (2010 ▶). For a related structure, see: Wang *et al.* (2006 ▶). For the stability of the temperature controller used for the data collection, see: Cosier & Glazer (1986 ▶).
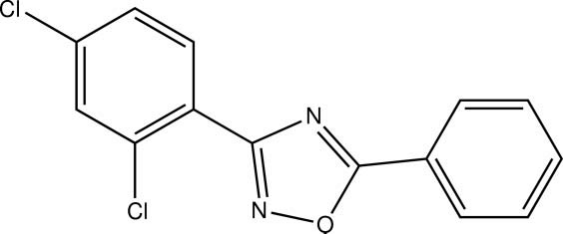


## Experimental

### 

#### Crystal data

C_14_H_8_Cl_2_N_2_O
*M* *_r_* = 291.12
Triclinic, 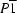

*a* = 3.8035 (2) Å
*b* = 10.9666 (7) Å
*c* = 14.6949 (9) Å
α = 99.044 (2)°
β = 91.158 (2)°
γ = 98.891 (2)°
*V* = 597.43 (6) Å^3^
*Z* = 2
Mo *K*α radiation
μ = 0.53 mm^−1^
*T* = 100 K
0.41 × 0.13 × 0.09 mm

#### Data collection

Bruker SMART APEXII CCD area-detector diffractometer
Absorption correction: multi-scan (*SADABS*; Bruker, 2005 ▶) *T*_min_ = 0.809, *T*_max_ = 0.953
12084 measured reflections
2692 independent reflections
2355 reflections with *I* > 2σ(*I*)
*R*_int_ = 0.035

#### Refinement

*R*[*F* ^2^ > 2σ(*F* ^2^)] = 0.035
*wR*(*F* ^2^) = 0.121
*S* = 1.14
2692 reflections
204 parameters
All H-atom parameters refined
Δρ_max_ = 0.55 e Å^−3^
Δρ_min_ = −0.39 e Å^−3^

### 

Data collection: *APEX2* (Bruker, 2005 ▶); cell refinement: *SAINT* (Bruker, 2005 ▶); data reduction: *SAINT*; program(s) used to solve structure: *SHELXTL* (Sheldrick, 2008 ▶); program(s) used to refine structure: *SHELXTL*; molecular graphics: *SHELXTL*; software used to prepare material for publication: *SHELXTL* and *PLATON* (Spek, 2009 ▶).

## Supplementary Material

Crystal structure: contains datablocks global, I. DOI: 10.1107/S160053681000927X/sj2743sup1.cif

Structure factors: contains datablocks I. DOI: 10.1107/S160053681000927X/sj2743Isup2.hkl

Additional supplementary materials:  crystallographic information; 3D view; checkCIF report

## Figures and Tables

**Table 1:** Hydrogen-bond geometry (Å, °)

*D*—H⋯*A*	*D*—H	H⋯*A*	*D*⋯*A*	*D*—H⋯*A*
C3—H3*A*⋯Cl1^i^	0.95 (3)	2.81 (3)	3.577 (2)	138.6 (19)

Symmetry code: (i) 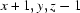.

**Fig. 1.**
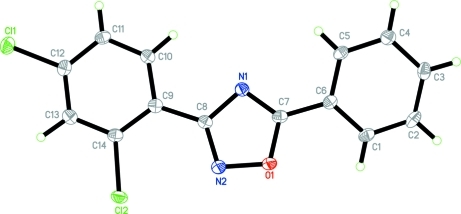
The molecular structure of (I), showing 50% probability displacement ellipsoids and the atom-numbering scheme.

**Fig. 2.**
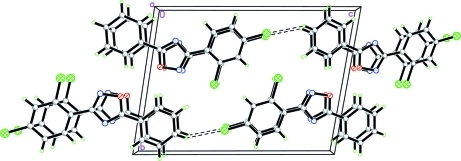
The crystal structure of (I), showing infinite chains along the c-axis.

## supplementary crystallographic information

### Comment

Heterocyclic compounds are becoming increasingly important in recent years due to their pharmacological activities (Isloor *et al.* 2010). Nitrogen- and oxygen-containing five/six membered heterocyclic compounds are of enormous significance in the field of medicinal chemistry (Chandrakantha *et al.*, 2010). Oxadiazoles play a very vital role in the preparation of various biologically active drugs with anti-inflammatory (Andersen *et al.*, 1994), anti-cancer (Showell *et al.*, 1991), anti-HIV (Watjen *et al.*, 1989), anti-diabetic and anti-microbial (Swain *et al.*, 1991) activities. The results of biological studies showed that oxadiazole derivatives also possess maximum anti-inflammatory, analgesic and minimum ulcerogenic and lipid per-oxidation (Clitherow *et al.*, 1996) properties.

The geometrical parameters of (I) are within the normal range and comparable with those for a related structure (Wang *et al.*, 2006). The mean plane of of the oxadiazole ring (C7/C8/N1/N2/O1) is almost coplanar with the C9-C14 benzene ring [dihedral angle = 2.3 (1)°] but slightly twisted with the C1-C6 benzene ring [dihedral angle = 9.5 (1)°].

The C-H···Cl (Table 1) interactions link the molecules into infinite chains along the c-axis and these chains are stacked along the a-axis.

### Experimental

The title compound was prepared by heating a solution of 2,4-dichloro- N'-hydroxy-benzamidine (1 g,0.0042 mol) and benzoylchloride (0.65 g.0.004 mol) in pyridine (30 ml). The reaction mixture was heated at 114 °C for 1.5 hour and concentrated under vacuum. Further purification was done by column chromatography. The solid obtained was recrystalised using dichloromethane.Yield: 1 g (76%),  Melting point 413-415 K.

### Refinement

All H atoms were located in a difference Fourier map and refined freely.

### Crystal data

**Table:**

C_14_H_8_Cl_2_N_2_O	*Z* = 2
*M**_r_* = 291.12	*F*(000) = 296
Triclinic, *P*1	*D*_x_ = 1.618 Mg m^−^^3^
Hall symbol: -P 1	Mo *K*α radiation, λ = 0.71073 Å
*a* = 3.8035 (2) Å	Cell parameters from 7625 reflections
*b* = 10.9666 (7) Å	θ = 2.5–36.0°
*c* = 14.6949 (9) Å	µ = 0.53 mm^−^^1^
α = 99.044 (2)°	*T* = 100 K
β = 91.158 (2)°	Block, colourless
γ = 98.891 (2)°	0.41 × 0.13 × 0.09 mm
*V* = 597.43 (6) Å^3^	

### Data collection

**Table:**

Bruker SMART APEXII CCD area-detector diffractometer	2692 independent reflections
Radiation source: fine-focus sealed tube	2355 reflections with *I* > 2σ(*I*)
graphite	*R*_int_ = 0.035
φ and ω scans	θ_max_ = 27.5°, θ_min_ = 1.4°
Absorption correction: multi-scan (*SADABS*; Bruker, 2005)	*h* = −4→4
*T*_min_ = 0.809, *T*_max_ = 0.953	*k* = −14→14
12084 measured reflections	*l* = −19→19

### Refinement

**Table:**

Refinement on *F*^2^	Primary atom site location: structure-invariant direct methods
Least-squares matrix: full	Secondary atom site location: difference Fourier map
*R*[*F*^2^ > 2σ(*F*^2^)] = 0.035	Hydrogen site location: inferred from neighbouring sites
*wR*(*F*^2^) = 0.121	All H-atom parameters refined
*S* = 1.14	*w* = 1/[σ^2^(*F*_o_^2^) + (0.0727*P*)^2^ + 0.3227*P*] where *P* = (*F*_o_^2^ + 2*F*_c_^2^)/3
2692 reflections	(Δ/σ)_max_ = 0.001
204 parameters	Δρ_max_ = 0.55 e Å^−^^3^
0 restraints	Δρ_min_ = −0.39 e Å^−^^3^

### Special details

**Table:**

Experimental. The crystal was placed in the cold stream of an Oxford Cyrosystems Cobra open-flow nitrogen cryostat (Cosier & Glazer, 1986) operating at 100.0 (1) K.
Geometry. All esds (except the esd in the dihedral angle between two l.s. planes) are estimated using the full covariance matrix. The cell esds are taken into account individually in the estimation of esds in distances, angles and torsion angles; correlations between esds in cell parameters are only used when they are defined by crystal symmetry. An approximate (isotropic) treatment of cell esds is used for estimating esds involving l.s. planes.
Refinement. Refinement of F^2^ against ALL reflections. The weighted R-factor wR and goodness of fit S are based on F^2^, conventional R-factors R are based on F, with F set to zero for negative F^2^. The threshold expression of F^2^ > 2sigma(F^2^) is used only for calculating R-factors(gt) etc. and is not relevant to the choice of reflections for refinement. R-factors based on F^2^ are statistically about twice as large as those based on F, and R- factors based on ALL data will be even larger.

### Fractional atomic coordinates and isotropic or equivalent isotropic displacement parameters (Å^2^)

**Table:**

	*x*	*y*	*z*	*U*_iso_*/*U*_eq_	
Cl1	0.67894 (14)	0.17290 (4)	0.56305 (3)	0.02303 (17)	
Cl2	0.74585 (13)	0.52050 (4)	0.34575 (3)	0.01905 (16)	
O1	1.0808 (4)	0.40239 (12)	0.07273 (9)	0.0188 (3)	
N1	1.1293 (4)	0.22993 (14)	0.12866 (10)	0.0152 (3)	
N2	0.9704 (5)	0.42193 (15)	0.16440 (11)	0.0186 (3)	
C1	1.3840 (5)	0.31992 (18)	−0.09742 (13)	0.0182 (4)	
C2	1.5167 (6)	0.27272 (19)	−0.18064 (13)	0.0207 (4)	
C3	1.5623 (5)	0.14861 (19)	−0.19917 (13)	0.0197 (4)	
C4	1.4763 (5)	0.06975 (18)	−0.13435 (12)	0.0180 (4)	
C5	1.3476 (5)	0.11647 (17)	−0.05099 (12)	0.0157 (4)	
C6	1.3013 (5)	0.24188 (17)	−0.03204 (12)	0.0146 (4)	
C7	1.1707 (5)	0.28734 (17)	0.05746 (12)	0.0152 (4)	
C8	1.0052 (5)	0.31698 (17)	0.19311 (12)	0.0141 (4)	
C9	0.9255 (5)	0.28872 (16)	0.28577 (12)	0.0146 (4)	
C10	0.9670 (5)	0.16979 (17)	0.30360 (12)	0.0155 (4)	
C11	0.8962 (5)	0.13252 (18)	0.38802 (13)	0.0172 (4)	
C12	0.7795 (5)	0.21663 (18)	0.45703 (12)	0.0171 (4)	
C13	0.7382 (5)	0.33600 (18)	0.44337 (12)	0.0172 (4)	
C14	0.8094 (5)	0.37087 (16)	0.35794 (12)	0.0155 (4)	
H1A	1.360 (7)	0.402 (3)	−0.0859 (17)	0.026 (6)*	
H2A	1.572 (7)	0.323 (2)	−0.2233 (18)	0.024 (6)*	
H3A	1.664 (7)	0.121 (2)	−0.2558 (18)	0.029 (7)*	
H4A	1.483 (6)	−0.015 (2)	−0.1458 (15)	0.015 (5)*	
H5A	1.325 (7)	0.068 (2)	−0.0091 (17)	0.020 (6)*	
H10A	1.028 (7)	0.109 (2)	0.2564 (16)	0.019 (6)*	
H11A	0.922 (6)	0.054 (2)	0.3967 (15)	0.014 (5)*	
H13A	0.635 (7)	0.390 (2)	0.4924 (17)	0.022 (6)*	

### Atomic displacement parameters (Å^2^)

**Table:**

	*U*^11^	*U*^22^	*U*^33^	*U*^12^	*U*^13^	*U*^23^
Cl1	0.0276 (3)	0.0272 (3)	0.0163 (2)	0.0063 (2)	0.00286 (18)	0.00754 (18)
Cl2	0.0228 (3)	0.0169 (2)	0.0191 (2)	0.00957 (18)	−0.00111 (17)	0.00205 (17)
O1	0.0261 (8)	0.0160 (6)	0.0165 (6)	0.0086 (5)	0.0003 (5)	0.0045 (5)
N1	0.0149 (8)	0.0165 (7)	0.0148 (7)	0.0051 (6)	−0.0008 (6)	0.0025 (6)
N2	0.0233 (9)	0.0185 (8)	0.0160 (7)	0.0077 (6)	0.0014 (6)	0.0043 (6)
C1	0.0180 (10)	0.0182 (9)	0.0199 (9)	0.0044 (7)	−0.0031 (7)	0.0068 (7)
C2	0.0208 (10)	0.0256 (10)	0.0180 (9)	0.0034 (8)	−0.0013 (7)	0.0112 (7)
C3	0.0185 (10)	0.0277 (10)	0.0139 (8)	0.0062 (8)	−0.0005 (7)	0.0039 (7)
C4	0.0174 (10)	0.0194 (9)	0.0178 (8)	0.0050 (7)	−0.0031 (7)	0.0035 (7)
C5	0.0138 (9)	0.0173 (8)	0.0171 (8)	0.0031 (7)	−0.0019 (6)	0.0063 (7)
C6	0.0121 (9)	0.0171 (8)	0.0148 (8)	0.0026 (7)	−0.0031 (6)	0.0037 (6)
C7	0.0128 (9)	0.0153 (8)	0.0181 (8)	0.0037 (7)	−0.0037 (6)	0.0036 (6)
C8	0.0106 (9)	0.0157 (8)	0.0160 (8)	0.0035 (6)	−0.0028 (6)	0.0016 (6)
C9	0.0103 (9)	0.0176 (9)	0.0157 (8)	0.0023 (7)	−0.0034 (6)	0.0026 (6)
C10	0.0137 (9)	0.0166 (8)	0.0163 (8)	0.0040 (7)	−0.0016 (6)	0.0013 (6)
C11	0.0170 (10)	0.0163 (9)	0.0191 (8)	0.0032 (7)	−0.0021 (7)	0.0050 (7)
C12	0.0146 (9)	0.0222 (9)	0.0150 (8)	0.0027 (7)	−0.0016 (6)	0.0049 (7)
C13	0.0150 (9)	0.0203 (9)	0.0164 (8)	0.0050 (7)	−0.0024 (7)	0.0009 (7)
C14	0.0128 (9)	0.0157 (8)	0.0183 (8)	0.0036 (7)	−0.0029 (6)	0.0026 (7)

### Geometric parameters (Å, °)

**Table:**

Cl1—C12	1.7338 (18)	C4—H4A	0.92 (2)
Cl2—C14	1.7310 (18)	C5—C6	1.399 (3)
O1—C7	1.344 (2)	C5—H5A	0.87 (2)
O1—N2	1.413 (2)	C6—C7	1.457 (2)
N1—C7	1.303 (2)	C8—C9	1.470 (2)
N1—C8	1.380 (2)	C9—C10	1.401 (3)
N2—C8	1.311 (2)	C9—C14	1.405 (3)
C1—C2	1.387 (3)	C10—C11	1.385 (3)
C1—C6	1.394 (2)	C10—H10A	0.94 (2)
C1—H1A	0.91 (3)	C11—C12	1.388 (3)
C2—C3	1.384 (3)	C11—H11A	0.91 (2)
C2—H2A	0.91 (3)	C12—C13	1.387 (3)
C3—C4	1.395 (3)	C13—C14	1.389 (3)
C3—H3A	0.95 (3)	C13—H13A	0.99 (3)
C4—C5	1.381 (3)		
			
C7—O1—N2	106.71 (14)	O1—C7—C6	119.03 (16)
C7—N1—C8	102.57 (15)	N2—C8—N1	114.69 (16)
C8—N2—O1	103.00 (15)	N2—C8—C9	125.14 (17)
C2—C1—C6	119.53 (18)	N1—C8—C9	120.17 (15)
C2—C1—H1A	119.2 (16)	C10—C9—C14	117.16 (16)
C6—C1—H1A	121.2 (16)	C10—C9—C8	117.41 (16)
C3—C2—C1	120.30 (17)	C14—C9—C8	125.43 (16)
C3—C2—H2A	119.8 (16)	C11—C10—C9	122.41 (17)
C1—C2—H2A	119.9 (16)	C11—C10—H10A	116.9 (14)
C2—C3—C4	120.40 (18)	C9—C10—H10A	120.5 (14)
C2—C3—H3A	117.6 (16)	C10—C11—C12	118.35 (17)
C4—C3—H3A	122.0 (16)	C10—C11—H11A	120.0 (14)
C5—C4—C3	119.60 (18)	C12—C11—H11A	121.6 (14)
C5—C4—H4A	116.9 (14)	C13—C12—C11	121.57 (17)
C3—C4—H4A	123.3 (14)	C13—C12—Cl1	118.49 (15)
C4—C5—C6	120.14 (17)	C11—C12—Cl1	119.94 (15)
C4—C5—H5A	117.2 (16)	C12—C13—C14	118.95 (17)
C6—C5—H5A	122.2 (16)	C12—C13—H13A	118.8 (14)
C1—C6—C5	120.04 (17)	C14—C13—H13A	121.9 (14)
C1—C6—C7	121.86 (17)	C13—C14—C9	121.56 (17)
C5—C6—C7	118.10 (16)	C13—C14—Cl2	116.22 (14)
N1—C7—O1	113.04 (16)	C9—C14—Cl2	122.21 (14)
N1—C7—C6	127.93 (16)		
			
C7—O1—N2—C8	−0.24 (19)	C7—N1—C8—N2	0.1 (2)
C6—C1—C2—C3	0.8 (3)	C7—N1—C8—C9	−179.53 (16)
C1—C2—C3—C4	−0.2 (3)	N2—C8—C9—C10	177.93 (18)
C2—C3—C4—C5	−0.5 (3)	N1—C8—C9—C10	−2.5 (3)
C3—C4—C5—C6	0.5 (3)	N2—C8—C9—C14	−1.8 (3)
C2—C1—C6—C5	−0.9 (3)	N1—C8—C9—C14	177.79 (17)
C2—C1—C6—C7	178.25 (17)	C14—C9—C10—C11	0.4 (3)
C4—C5—C6—C1	0.2 (3)	C8—C9—C10—C11	−179.33 (17)
C4—C5—C6—C7	−178.94 (17)	C9—C10—C11—C12	0.1 (3)
C8—N1—C7—O1	−0.2 (2)	C10—C11—C12—C13	−0.9 (3)
C8—N1—C7—C6	179.12 (18)	C10—C11—C12—Cl1	178.69 (14)
N2—O1—C7—N1	0.3 (2)	C11—C12—C13—C14	1.2 (3)
N2—O1—C7—C6	−179.11 (15)	Cl1—C12—C13—C14	−178.38 (14)
C1—C6—C7—N1	−169.98 (19)	C12—C13—C14—C9	−0.7 (3)
C5—C6—C7—N1	9.1 (3)	C12—C13—C14—Cl2	179.14 (14)
C1—C6—C7—O1	9.4 (3)	C10—C9—C14—C13	−0.1 (3)
C5—C6—C7—O1	−171.53 (16)	C8—C9—C14—C13	179.63 (17)
O1—N2—C8—N1	0.1 (2)	C10—C9—C14—Cl2	−179.90 (14)
O1—N2—C8—C9	179.68 (16)	C8—C9—C14—Cl2	−0.2 (3)

### Hydrogen-bond geometry (Å, °)

**Table:**

*D*—H···*A*	*D*—H	H···*A*	*D*···*A*	*D*—H···*A*
C3—H3A···Cl1^i^	0.95 (3)	2.81 (3)	3.577 (2)	138.6 (19)

Symmetry codes: (i) *x*+1, *y*, *z*−1.
